# Efficacy of tumor-targeting *Salmonella typhimurium* A1-R in combination with anti-angiogenesis therapy on a pancreatic cancer patient-derived orthotopic xenograft (PDOX) and cell line mouse models

**DOI:** 10.18632/oncotarget.2641

**Published:** 2014-10-28

**Authors:** Yukihiko Hiroshima, Yong Zhang, Takashi Murakami, Ali Maawy, Shinji Miwa, Mako Yamamoto, Shuya Yano, Sho Sato, Masashi Momiyama, Ryutaro Mori, Ryusei Matsuyama, Takashi Chishima, Kuniya Tanaka, Yasushi Ichikawa, Michael Bouvet, Itaru Endo, Ming Zhao, Robert M. Hoffman

**Affiliations:** ^1^ AntiCancer, Inc., San Diego, CA, USA; ^2^ Department of Surgery, University of California San Diego, San Diego, CA, USA; ^3^ Yokohama City University Graduate School of Medicine, Yokohama, Japan

**Keywords:** Pancreatic cancer, Salmonella typhimurium A1-R, patient-derived orthotopic xenograft (PDOX), orthotopic, nude mice, GFP, VEGF, anti-angiogenic therapy, bevacizumab, gemcitabine

## Abstract

The aim of the present study was to examine the efficacy of tumor-targeting *Salmonella typhimurium* A1-R treatment following anti-vascular endothelial growth factor (VEGF) therapy on VEGF-positive human pancreatic cancer. A pancreatic cancer patient-derived orthotopic xenograft (PDOX) that was VEGF-positive and an orthotopic VEGF-positive human pancreatic cancer cell line (MiaPaCa-2-GFP) as well as a VEGF-negative cell line (Panc-1) were tested. Nude mice with these tumors were treated with gemcitabine (GEM), bevacizumab (BEV), and *S. typhimurium* A1-R. BEV/GEM followed by *S. typhimurium* A1-R significantly reduced tumor weight compared to BEV/GEM treatment alone in the PDOX and MiaPaCa-2 models. Neither treatment was as effective in the VEGF-negative model as in the VEGF-positive models. These results demonstrate that *S. typhimurium* A1-R following anti-angiogenic therapy is effective on pancreatic cancer including the PDOX model, suggesting its clinical potential.

## INTRODUCTION

Pancreatic cancer is one of the most aggressive malignant tumors with a 23 % 1-year survival rate and < 2 % 5-year survival rate. The two most commonly used chemotherapy drugs approved for the treatment of pancreatic cancer are gemcitabine (GEM) and 5-ﬂuorouracil (5-FU). In recent years, little progress has been made in understanding and treatment of this disease [1].

In pancreatic cancer, overexpression of vascular endothelial growth factor (VEGF) and its receptors is associated with poor prognosis and increased metastatic potential [2, 3]. Bevacizumab (BEV) is a humanized monoclonal VEGF-neutralizing antibody that many tumors become resistant to after a short period of response [4].

Our laboratory has previously developed a genetically modified bacterial strain, *Salmonella typhimurium* A1, selected for anticancer activity *in vivo*. *S. typhimurium* A1 is auxotrophic (leu/arg-dependent) [5]. The strain targets and grows in tumors. In contrast, normal tissue is cleared of these bacterial even in immunodeficient athymic mice. In order to increase the tumor-targeting capability of A1, the strain was re-isolated after infection of a human colon tumor growing in nude mice. The tumor-isolated strain, termed *S. typhimurium* A1-R, had increased targeting for cells *in vivo* as well as *in vitro* [6].

*S. typhimurium* A1-R is effective against prostate cancer [7], breast cancer [6, 8], pancreatic cancer [9-12], glioma [13, 14], lung cancer [15], fibrosarcoma [16] and osteosarcoma [17].

In the present study, we demonstrate the efficacy of *S. typhimurium* A1-R following antiangiogenic therapy with bevacizumab/gemcitabine (BEV/GEM) in patient-derived orthotopic xenograft (PDOX) and cell line nude-mouse models of pancreatic cancer.

## RESULTS AND DISCUSSION

### Differential expression patterns of VEGF-related genes in pancreatic cancer cell lines

**Figure 1:**
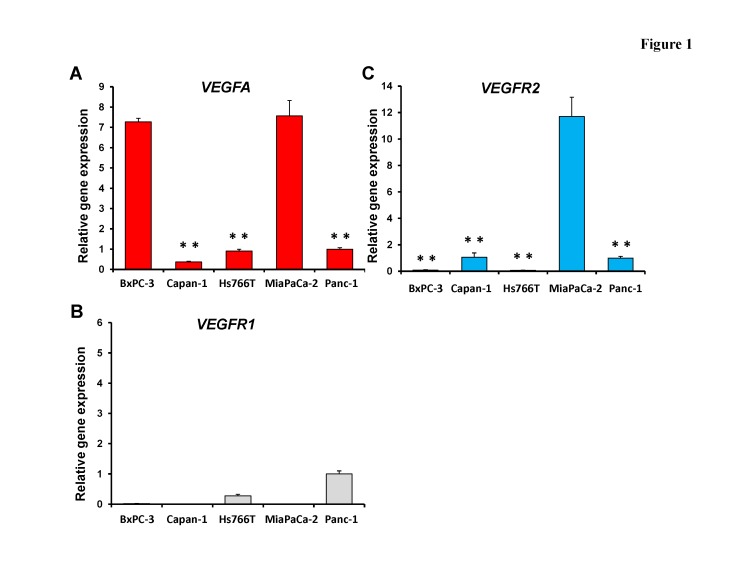
mRNA expression of *VEGFA* (A), *VEGFR1* (B) and *VEGFR2* (C) in pancreatic cancer cell lines mRNA expression was determined with real-time RT-PCR. MiaPaCa-2 significantly expressed *VEGFA* more than other cell lines (p < 0.001) except for BxPC-3 (p = 0.558) (A). MiaPaCa-2 significantly expressed *VEGFR2* more than other the cell lines (BxPC-3: p = 0.005, Capan-1: p < 0.001; Hs766T: p = 0.005; and Panc-1: p = 0.006) (C). *VEGFR1* expression was not detected in MiaPaCa-2 and Capan-1 cell lines (B). Data for each treatment are represented as the mean ± SD. ** p < 0.01.

In order to identify potential BEV-sensitive pancreatic cancer cell lines, mRNA expression of *VEGFA*, *VEGFR1* and *VEGFR2* in pancreatic cancer cell lines (BxPC-3, Capan-1, Hs766T, MiaPaCa-2 and Panc-1) was examined by real-time RT-PCR (Fig. 1). MiaPaCa-2 expressed *VEGFA* significantly more than other cell lines (p < 0.001) except for BxPC-3 (p = 0.558) (Fig. 1A). MiaPaCa-2 expressed *VEGFR2* significantly more than other cell lines (BxPC-3: p = 0.005; Capan-1: p < 0.001; Hs766T: p = 0.005; and Panc-1: p = 0.006) (Fig. 1C). *VEGFR1* expression was not detected in MiaPaCa-2 and Capan-1 cell lines (Fig. 1B).

### *S. typhimurium* A1-R killed MiaPaCa-2 and Panc-1 pancreatic cancer cells *in vitro*

**Figure 2:**
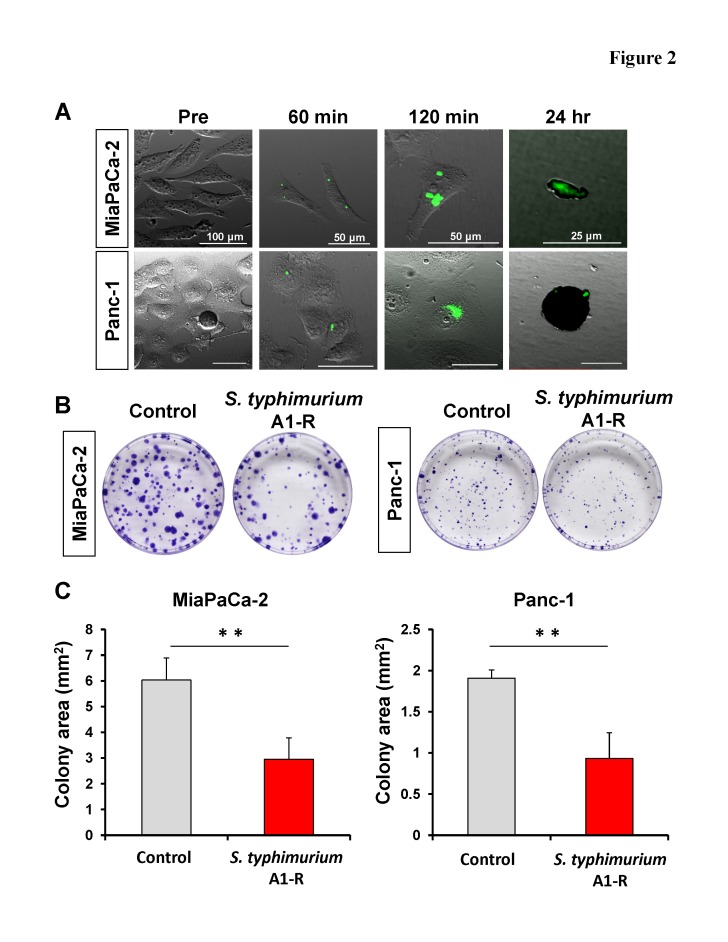
Efficacy of *S. typhimurium* A1-R on pancreatic cancer cell lines (A) Confocal imaging of MiaPaCa-2 and Panc-1 pancreatic cancer cells infected with *S. typhimurium* A1-R *in vitro*. *S. typhimurium* A1-R infection was detected in both pancreatic cancer cell types after 60 min. *S. typhimurium* A1-R replicated in the cells after 120 min. *S. typhimurium* A1-R showed the ability to infect and induce apoptosis in both cell types after 24 hr. Scale bars: 100 μm (pre); 50 μm (60 and 120 min); 25 μm (24 hr). (B and C) MiaPaCa-2 and Panc-1 were treated with *S. typhimurium* A1-R. Clonogenic assays show that *S. typhimurium* A1-R significantly reduced colony formation of both pancreatic cancer cell lines compared to the control groups *in vitro* (MiaPaCa-2: p = 0.001 and Panc-1: p < 0.001). ** p < 0.01.

GFP-expressing *S. typhimurium* A1-R invaded MiaPaCa-2 and Panc-1 pancreatic cancer cells as early as 60 min, and replicated in the cells 120 min after infection. Both cancer cell types appeared to die via apoptosis 24 hr after bacterial infection (Fig. 2A). In the clonogenic assay, the average colony area of MiaPaCa-2 treated with *S. typhimurium* A1-R was 2.95 ± 0.84 mm^2^ compared to the untreated control, 6.03 ± 0.86 mm^2^. The average colony area of Panc-1 treated with *S. typhimurium* A1-R was 0.93 ± 0.31 mm^2^, compared to the untreated control, 1.91 ± 0.10 mm^2^. *S. typhimurium* A1-R significantly reduced colony formation of both pancreatic cancer cell lines compared to the control (MiaPaCa-2: p = 0.001 and Panc-1: p < 0.001) (Fig. 2B and 2C).

### Differential sensitivity to BEV in pancreatic cancer cell lines growing subcutaneously in nude mice

**Figure 3:**
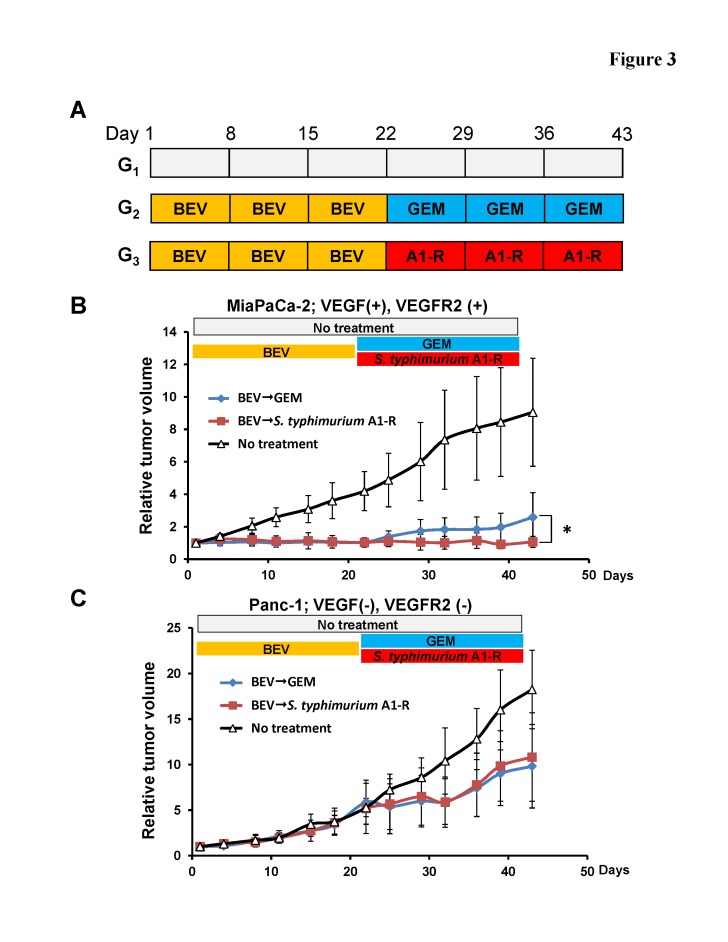
Efficacy of BEV treatment on pancreatic cancer cell lines which have different levels of *VEGFA* and *VEGFR2* expression (A) Schema of treatments on subcutaneous tumors. Based on RT-PCR results, MiaPaCa-2 was determined to be VEGFA-positive and VEGFR2-positive. Panc-1 was determined as VEGFA-negative and VEGFR2-negative. In order to determine the efficacy of BEV on pancreatic cancer cell lines with different levels of *VEGFA* and *VEGFR2* expression, subcutaneous tumors from MiaPaCa-2 and Panc-1 cells were grown in nude mice and randomized to 3 groups as described in the Materials and Methods. (B) BEV significantly reduced the growth of the MiaPaCa-2 tumor compared to the control on Day 22 (p < 0.001). Both BEV → GEM and BEV → *S. typhimurium* A1-R treatments significantly reduced MiaPaCa-2 tumor growth compared to the control group on Day 43 (p = 0.001). BEV → *S. typhimurium* A1-R significantly reduced the MiaPaCa-2 tumor growth compared to BEV → GEM (p = 0.037). (C) BEV did not reduce the tumor growth of Panc-1 compared to the control on Day 22, but both BEV → GEM and BEV → *S. typhimurium* A1-R treatment significantly reduced tumor growth compared to the control on Day 43 (BEV → GEM: p = 0.023; BEV → *S. typhimurium* A1-R: p = 0.026). * p < 0.05.

Real-time RT-PCR of VEGF-related gene expression (Fig. 1) predicted that MiaPaCa-2 was BEV-sensitive and Panc-1 was BEV-resistant. The efficacy of BEV on these cell lines was first determined using a subcutaneous tumor mouse model (Fig. 3). The average tumor volume of the MiaPaCa-2 tumors treated with BEV was 1.04 ± 0.24 mm^3^ compared to the control which was 4.19 ± 1.21 mm^3^ on Day 22. The average tumor volume of the Panc-1 tumors treated with BEV was 5.50 ± 2.62 mm^3^ compared to the control which was 5.28 ± 0.99 mm^3^ on Day 22. BEV significantly reduced the growth of MiaPaCa-2 compared to the untreated controll group on Day 22 (p < 0.001) but did not reduce the growth of Panc-1 (Fig. 3).

### Efficacy of BEV on microvessel density in pancreatic cell lines growing subcutaneously in mice

**Figure 4:**
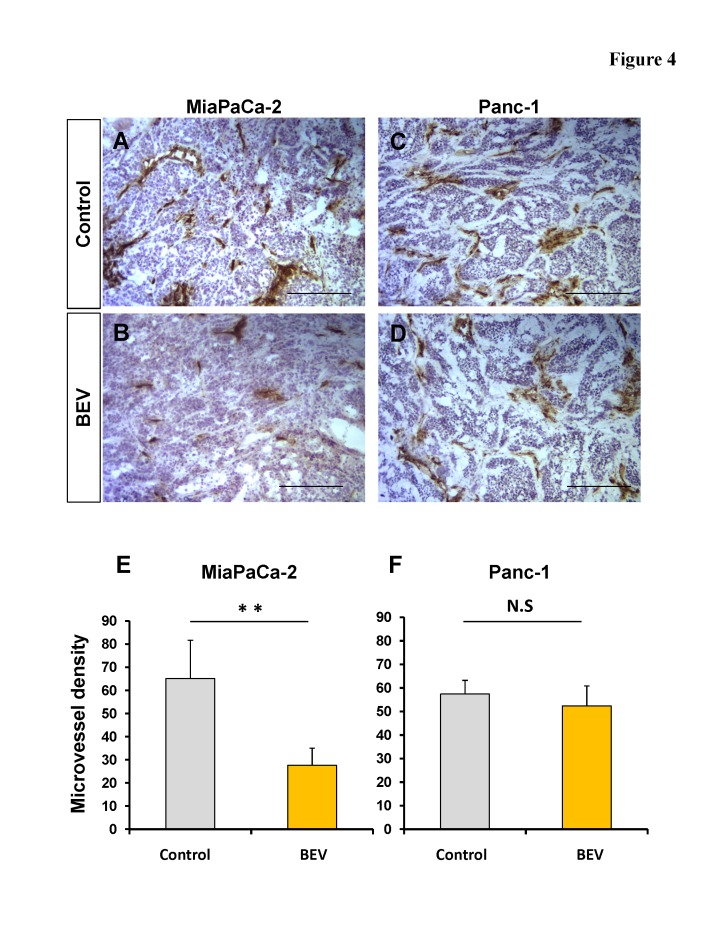
Microvessel density (MVD) in s.c. pancreatic cancer xenografts in nude mice treated with bevacizumab (BEV) To determine MVD, sections were stained with an antibody to CD31 as described in the Materials and Methods. MVD was determined by counting the number of CD31-positive vessels in three fields at ×100 magnification of the highest vascular density. The values are the average numbers of microvessels ± S.D. (bars) of five different tumors. (A-D) Representative images of frozen sections stained with anti-CD31 antibody. The number of vessels in MiaPaCa-2 tumors treated with BEV (B) was reduced compared to the control (A). In contrast, there was no difference between Panc-1 tumors treated with BEV (D) and control (C). Scale bars: 200 μm. (E and F) Bar graphs of MVD in pancreatic tumors with and without BEV treatment. BEV significantly reduced the MVD of MiaPaCa-2 compared to the control (p = 0.002) (E), but did not reduce the MVD of Panc-1 (F). ** p < 0.01.

Subcutaneous tumors (MiaPaCa-2 or Panc-1) were treated with BEV (5 mg/kg, twice a week for 2 weeks) and tumor samples were removed 7 days after the last treatment. Frozen sections from each tumor were stained with anti-mouse CD31 antibody, and the MVD was determined by counting three fields at ×100 magnification of the highest vascular density. The average MVD of the MiaPaCa-2 tumors treated with BEV was 27.6 ± 7.45 compared to the control which was 65.1 ± 16.5. The average MVD of the Panc-1 tumors treated with BEV was 52.4 ± 8.43 compared to the control which was 57.4 ± 5.81. BEV significantly reduced the MVD of the MiaPaCa-2 tumor compared to the control (p = 0.002) (Fig. 4A, B, E) but did not significantly reduce MVD of the Panc-1 tumor (Fig. 4C, D, F). These results are consistent with the expression levels of VEGF-related genes (Fig. 1), indicating that MiaPaCa-2 is BEV-sensitive and Panc-1 is BEV-resistant.

### Efficacy of *S. typhimurium* A1-R following BEV treatment on growth of BEV-sensitive and -resistant tumors

For the BEV-sensitive MiaPaCa-2 tumor, both BEV → GEM and BEV → *S. typhimurium* A1-R significantly reduced tumor growth compared to the controls on Day 43 (p = 0.001). BEV→*S. typhimurium* A1-R significantly reduced tumor growth compared to BEV → GEM (p = 0.037) (Fig. 3B). *S. typhimurium* A1-R completely suppressed growth of MiaPaCa-2 after BEV treatment (Fig. 3B). Both BEV → GEM and BEV →*S. typhimurium* A1-R significantly reduced tumor growth of the BEV-resistant tumor Panc-1 compared to the control (BEV → GEM: p = 0.023; BEV →*S. typhimurium* A1-R: p = 0.026) but to a lesser extent than MiaPaCa-2 (Fig. 3C). There was no significant difference in tumor growth inhibition between BEV →*S. typhimurium* A1-R and BEV →GEM.

**Figure 5:**
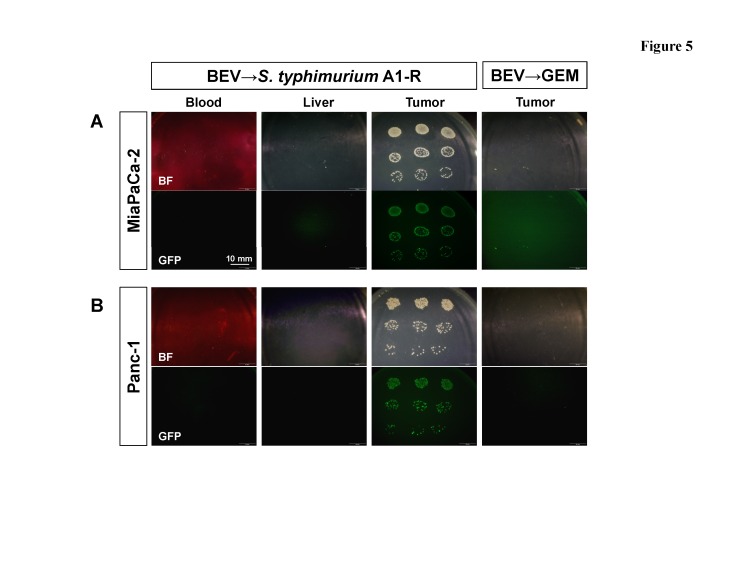
Distribution of GFP-labeled *S. typhimurium* A1-R bacteria in tumors and organs Livers and tumors were minced and mixed with PBS, as was blood. The PBS, was plated on LB agar to identify *S. typhimurium* A1-R in each tissue. Fluorescent *S. typhimurium* A1-R GFP colonies were observed with the OV100 Small Animal Imaging System (Olympus Corp., Tokyo, Japan). Representative images of GFP-labeled *S. typhimurium* A1-R cultured from the tumor and the normal organs (blood and liver) of the mice in the BEV → *S. typhimurium* A1-R and BEV → GEM groups. GFP-labeled *S. typhimurium* A1-R bacteria were clearly detected in both MiaPaCa-2 tumors (A) and Panc-1 tumors (B) in the BEV → *S. typhimurium* A1-R groups. No GFP-labeled *S. typhimurium* A1-R was detected in other tissues. Scale bars: 10 mm. BF= brightfield

Furthermore, GFP-labeled *S. typhimurium* A1-R was detected in the MiaPaCa-2 tumor after BEV → *S. typhimurium* A1-R treatment (Fig. 5). Our data suggest that *S. typhimurium* A1-R is able to survive and multiply even in the hypo-vascular area of the tumor treated with BEV and cause tumor shrinkage.

**Figure 6:**
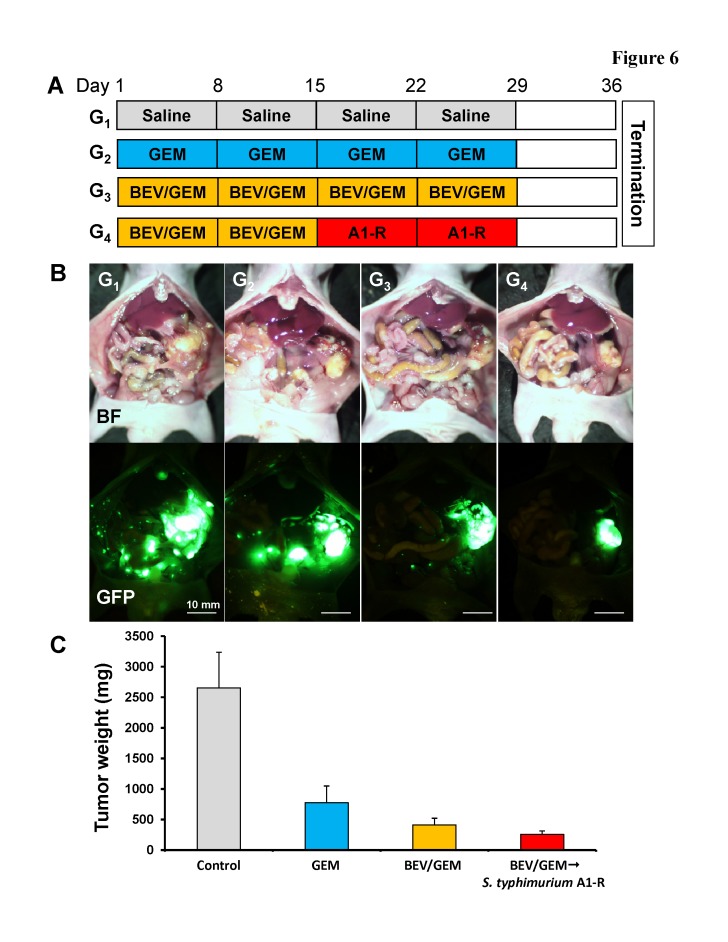
*S. typhimurium* A1-R treatment following BEV and GEM combination therapy of the MiaPaCa-2-GFP tumor in an orthotopic mouse model (A) Schema of treatment of orthotopic MiaPaCa-2-GFP tumors. After confirmation of tumor growth by imaging with the OV100, the mice with orthotopic tumors were randomized to 4 groups: (G_1_) saline (vehicle/control, 4 weeks); (G_2_) GEM (4 weeks); (G_3_) BEV (4 weeks) / GEM (4 weeks); and (G_4_) BEV (2 weeks) / GEM (2 weeks) → *S. typhimurium* A1-R (2 weeks). Animals underwent laparotomy on day 36, and the tumors were imaged with the OV100 and weighed and harvested for analysis. (B) Representative images at laparotomy. Upper panels indicate bright-field images (BF) and lower panels indicate GFP fluorescence images. A large primary tumor and many metastases spreading over the entire abdominal cavity were detected in the control group. Many metastases were found in the mice treated with GEM but rarely found in the BEV/GEM and BEV/GEM → *S. typhimurium* A1-R groups. All regimens significantly reduced tumor weight compared to the untreated control group (G_2_: p < 0.001; G_3_: p < 0.001; G_4_: p = 0.001). BEV/GEM significantly reduced tumor weight compared to GEM (p = 0.038). BEV/GEM → *S. typhimurium* A1-R significantly reduced tumor weight compared to GEM (p = 0.012) and BEV/GEM (p = 0.029)

To further demonstrate the advantages of *S. typhimurium* A1-R treatment following anti-VEGF therapy in the orthotopic mouse model, 20 mice with MiaPaCa-2-GFP orthotopic tumors were established and randomized to 4 groups: (G_1_) saline (vehicle/control, 4 weeks); (G_2_) GEM (4 weeks); (G_3_) BEV (4 weeks) / GEM (4 weeks) and (G_4_) BEV (2 weeks) / GEM (2 weeks) → *S. typhimurium* A1-R (2 weeks). Tumors were imaged and weighed after treatment (Fig. 6A). A large primary tumor and many metastases spreading over the entire abdominal cavity occurred in the control group (Fig. 6B; G_1_). Many metastases were found in the mice treated with GEM (G_2_) (Fig. 6B), but were rarely found in the BEV/GEM (G_3_) (Fig. 6B) and BEV/GEM → *S. typhimurium* A1-R groups (G_4_) (Fig. 6B; G_4_). The mean tumor weight of each group in the MiaPaCa-2-GFP model was as follows: (G_1_) Control: 2655.4 ± 583.9 mg; (G_2_) GEM: 775.9 ± 273.8 mg; (G_3_) BEV/GEM: 413.5 ± 108.3 mg; (G_4_) BEV/GEM → *S. typhimurium* A1-R: 257.5 ± 57.1 mg. All regimens significantly reduced tumor weight compared to the control group (G_2_: p < 0.001; G_3_: p < 0.001; G_4_: p = 0.001). BEV/GEM significantly reduced tumor weight compared to GEM (p = 0.038). BEV/GEM →*S. typhimurium* A1-R significantly reduced the tumor weight compared to GEM (p = 0.012) and BEV/GEM (p = 0.029). These results demonstrate that BEV/GEM → *S. typhimurium* A1-R sequential combination therapy is more effective than the BEV/GEM combination.

### *S. typhimurium* A1-R following anti-VEGF therapy is effective in a VEGF-positive PDOX model

Our laboratory pioneered surgical orthotopic implantation (SOI) metastatic mouse models of patient tumor specimens in the early 1990s [18-24]. These patient-derived orthotopic xenograft (PDOX) mouse models are more patient-like, especially with regard to metastasis, than ectopic subcutaneous models [25, 26].

**Figure 7:**
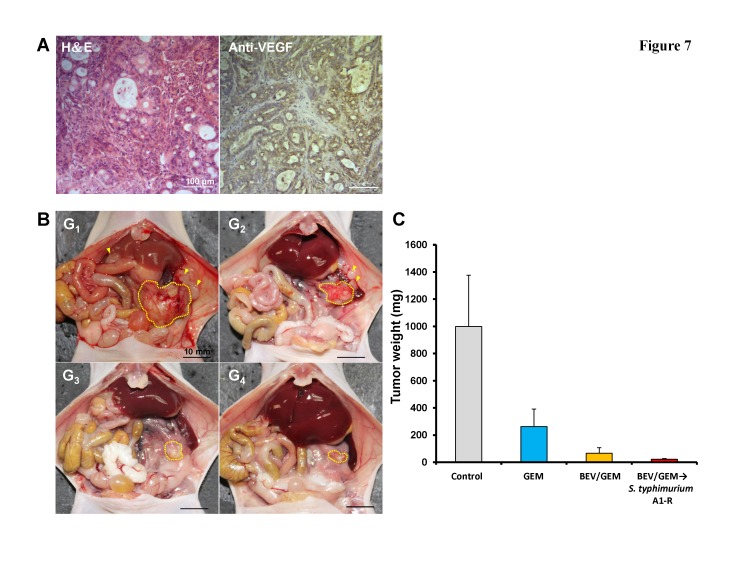
*S. typhimurium* A1-R treatment following BEV and GEM combination therapy on a VEGF-positive pancreatic cancer PDOX (A) Histological characterization of the PDOX. The PDOX was diagnosed as moderately-differentiated adenocarcinoma with H&E staining, and was strongly stained with an anti-VEGF antibody. Scale bars: 100 μm. (B) Representative images at laparotomy. The areas surrounded by the yellow broken lines indicate the primary tumors, and yellow arrow heads indicate metastasis. A large primary tumor and several metastases were detected in the control group (G_1_). A few metastases were found in the mice treated with GEM (G_2_) but rarely found in the BEV/GEM (G_3_) and BEV/GEM → *S. typhimurium* A1-R groups (G_4_). (C) Bar graphs of the total tumor weight (primary + metastasis) in each group. All regimens significantly reduced the tumor weight compared to the control group (G_2_: p = 0.004; G_3_: p = 0.002; G_4_: p = 0.001). BEV/GEM significantly reduced tumor weight compared to GEM (p = 0.005). BEV/GEM → *S. typhimurium* A1-R significantly reduced the tumor weight compared to GEM (p = 0.001) and BEV/GEM (p = 0.029)

A pancreatic cancer PDOX model was used to determine the efficacy of *S. typhimurium* A1-R treatment following anti-VEGF therapy. The patient tumor was a moderately-differentiated adenocarcinoma which expressed VEGF (Fig. 7A). Twenty mice with PDOXs were established and randomized to 4 groups and treated in the same way as the GFP MiaPaCa-2 orthotopic model. A large primary tumor and some metastases occurred in the control group. A few metastases were found in the mice treated with GEM but rarely in the BEV/GEM and BEV/GEM → *S. typhimurium* A1-R groups (Fig. 7B). The mean tumor weight of each group in the PDOX model was as follows: (G_1_) Control: 998.8 ± 377.7 mg; (G_2_) GEM: 263.1 ± 129.1 mg; (G_3_) BEV/GEM: 65.9 ± 41.9 mg; (G_4_) BEV/GEM →*S. typhimurium* A1-R: 21.9 ± 6.2 mg. All regimens significantly reduced tumor weight compared to the control group (G_2_: p = 0.004; G3: p = 0.002; G4: p = 0.001). BEV/GEM significantly reduced tumor weight compared to GEM (p = 0.005). BEV/GEM →*S. typhimurium* A1-R significantly reduced the tumor weight compared to GEM (p = 0.001) and BEV/GEM (p = 0.029). The results demonstrated in the PDOX model, BEV/GEM → *S. typhimurium* A1-R sequential combination therapy was also more effective than the BEV/GEM combination.

Previously developed concepts and strategies of highly selective tumor-targeting [27-34] can take advantage of the *S. typhimuium* A1-R tumor targeting described in the present report.

## MATERIALS AND METHODS

### Cell culture and establishment of a green fluorescent protein-labeled cancer cell line

Human pancreatic cancer cell lines Panc-1, MiaPaCa-2 and Hs766T were maintained in Dulbecco's modified Eagle's medium (Invitrogen, Carlsbad, CA, USA). Human pancreatic cancer cell lines BxPC-3 and Capan-1 were maintained in RPMI-1640 (Invitrogen, Carlsbad, CA, USA). All cell lines were incubated at 37°C in a humidified atmosphere containing 5% CO_2_. Each medium was supplemented with 10% fetal bovine serum, streptomycin, and penicillin (complete medium). The cells were collected after trypsinization and stained with trypan blue (Sigma-Aldrich, St. Louis, MO). Only viable cells which excluded trypan blue were counted with a hemocytometer (Hausser Scientific, Horsham, PA). For GFP gene transduction of cancer cells, 70% confluent human pancreatic cancer (MiaPaCa-2) cells were used. In brief, cells were incubated with a 1:1 precipitated mixture of retroviral supernatants of PT67-GFP packaging cells which express the GFP gene linked to the G418 resistance gene and RPMI 1640 (Irvine Scientific, Santa Ana, CA) containing 10% fetal bovine serum (FBS) (Hyclone Laboratories, Logan, UT) for 72 h. Fresh medium was replenished at this time. Cells were harvested with trypsin/EDTA 72 h post-transduction and subcultured at a ratio of 1:15 into medium, which contained 200 μg/ml of the selective agent G418. The level of G418 was increased stepwise up to 800 μg/ml [35, 36].

### Real-time RT-PCR

Total RNA was extracted from human pancreatic cancer cell lines (BxPC-3, Capan-1, Hs766T, MiaPaCa-2 and Panc-1) using TRIzol (Invitrogen, Carlsbad, CA, USA), followed by on-column clean up with the RNA spin mini kit (GE Healthcare BioSciences, Little Chalfont, UK). Total RNA (2 μg) was reverse transcribed using the High Capacity RNA-to-cDNA kit (Applied Biosystems, Foster City, CA, USA) for complementary DNA (cDNA) synthesis. cDNA (2 μl) in a final volume of 20 μl,was amplified using the following Taqman Gene Expression assays (Applied Biosystems): *VEGFA* (Hs00900055_m1); *VEGFR1* (Hs01052961_m1)*; VEGFR2* (Hs00911700_m1); and *glyceraldehyde-3-phosphate dehydrogenase* (*GAPDH*) endogenous control (Hs99999905_m1). All reactions were performed in triplicate using ABI 7900 HT Fast (Applied Biosystems). Analysis of relative gene expression data used the ΔΔCt method. An example is shown below:

Relative expression = 2 ^−ΔΔCt^, ΔΔCt = (Ct, _Target_ - Ct, _GAPDH_ ) - (Ct, _Panc-1_ - Ct, _GAPDH_ ).

### Preparation of bacteria

*S. typhimurium* A1-R was grown overnight on LB medium and then diluted 1:10 in LB medium. Bacteria were harvested at late-log phase, washed with PBS, and then diluted in PBS for use in experiments [6].

### Confocal imaging of cancer cells infected with *S. typhimurium* A1-R *in vitro*

Both MiaPaCa-2 and Panc-1 pancreatic cancer cell lines were infected with *S. typhimurium* A1-R GFP *in vitro*. Pancreatic cancer cells were grown in 24-well tissue culture plates to a density of approximately 10^4^ cells/well. *S. typhimurium* A1-R GFP were grown to late log in LB broth, diluted in cell culture medium and added to the cancer cells (1×10^7^ CFU/ml) and incubated at 37°C. After 40 min, the cells were rinsed and cultured in medium containing gentamycin sulfate (20 μg/ml) to kill external, but not internal bacteria. The interaction of *S. typhimurium* A1-R GFP with cancer cells *in vitro* was observed with confocal microscopy (Fluoview FV1000, Olympus, Tokyo, Japan). The excitation source was a semiconductor laser at 473 nm for GFP. Fluorescence images were obtained using the 20x/1.0 XLUMPLFLN objective [37].

### Clonogenic assay

MiaPaCa-2 and Panc-1 cells (1×10^3^ ) were seeded in 35 mm dishes. *S. typhimurium* A1-R (1×10^7^ CFU/ml) was added to the cancer cells and incubated at 37°C. After 40 min, the cells were rinsed and cultured in medium containing gentamycin sulfate (20 μg/ml). After 7-days culture, the cancer-cell colonies were fixed with ethanol and then stained with crystal violet. ImageJ was used to quantify the cell colonies.

### Animals

Male athymic (*nu/nu*) nude mice (AntiCancer, Inc., San Diego) (4-6 weeks) were used in this study. Mice were kept in a barrier facility under HEPA filtration. Mice were fed with autoclaved laboratory rodent diet. All surgical procedures and imaging were performed with the animals anesthetized by intramuscular injection of a solution of 50% ketamine, 38% xylazine, and 12% acepromazine maleate (0.02 ml). All animal studies were conducted in accordance with the principals and procedures outlined in the NIH Guide for the Care and Use of Laboratory Animals under PHS Assurance Number A3873-1.

### Subcutaneous pancreatic cancer cell implantation

Panc-1 and MiaPaCa-2 pancreatic cancer cells were harvested by trypsinization and washed twice with serum-free medium. Cells (2×10^6^ in 100 μl serum-free medium) were injected subcutaneously within 30 min of harvesting, over the right and left flanks in male nude mice. Subcutaneous tumors were allowed to grow for 2-4 weeks until large enough for subsequent experiments or orthotopic implantation.

### Assessment of microvessel density (MVD) in xenograft tumors

Frozen tumor sections (7 μm) were fixed with methanol. The sections were then treated for 30 min with hydrogen peroxide (0.3%) to block endogenous peroxidase activity. After incubation with normal goat serum 15%, the sections were incubated with anti-mouse CD31 (1:100; BD Pharmigen, San Jose, CA, USA) for 1 hour at room temperature. The primary antibodies were detected using anti-rat secondary antibodies and avidin/biotin/horseradish peroxidase complex (Vector Laboratories, Burlingame, CA, USA) for 30 min at room temperature. The labeled antigens were visualized with the DAB kit (DAKO Cytomation, Kyoto, Japan). The sections were counterstained with hematoxylin and examined using a BH-2 microscope (Olympus) equipped with an INFINITY1 2.0 megapixel CMOS digital camera (Lumenera Corporation, Ottawa, Canada). All images were acquired using INFINITY ANALYZE software (Lumenera Corporation) without post-acquisition processing. MVD was determined by counting three fields at ×100 magnification of the highest vascular density.

### Specimen collection

Patient pancreatic tumor samples were procured with informed written consent and the study was conducted under the approval of the Institutional Review Board of the UC San Diego Medical Center.

### Orthotopic tumor implantation

A small transverse incision (6- to 10-mm) was made on the left flank of the mouse through the skin and peritoneum. The tail of the pancreas was exposed through this incision, and a single tumor fragment (3-mm^3^.) harvested from a subcutaneous tumor was sutured to the tail of the pancreas using 8-0 nylon surgical sutures (Ethilon; Ethicon Inc., NJ, USA). On completion, the tail of the pancreas was returned to the abdomen, and the incision was closed in one layer using 6-0 nylon surgical sutures [19, 24].

### Treatment

Mice were randomized to 4 groups and treated as follows: (G_1_) saline (vehicle/control, ip, weekly, 4 weeks); (G_2_) GEM (80 mg/kg, ip, weekly, 4 weeks); (G_3_) BEV (5 mg/kg, ip, twice a week, 4 weeks) / GEM (Eli Lilly, Indianapolis, IN) (80 mg/kg, ip, weekly, 4 weeks) and (G_4_) BEV (Genentech, South San Francisco, CA) (5 mg/kg, ip, twice a week, 2 weeks) / GEM (80 mg/kg, ip, weekly, 2 weeks) → *S. typhimurium* A1-R (5 × 10^7^ CFU/body, iv, weekly, 2 weeks) (Fig. 4A). Each treatment group comprised 5 tumor-bearing mice. Body weights of the mice were determined on a balance once a week. Tumor size was measured with calipers in the subcutaneous models and at laparotomy in the orthotopic models on day 36. Tumors were imaged with an OV100 Small Animal Imaging System (Olympus, Tokyo, Japan) or a Canon EOS 60D digital camera with an EF–S18–55 IS lens (Canon, Tokyo, Japan) and weighed and harvested for analysis.

### Tissue histology

Tumor samples were removed with surrounding normal tissues at the time of resection. Fresh tissue samples were fixed in 10% formalin and embedded in paraffin before sectioning and staining. Tissue sections (3 μm) were deparaffinized in xylene and rehydrated in an ethanol series. Hematoxylin and eosin (H & E) staining was performed according to standard protocols.

### Fluorescence *in vivo* imaging

The OV100 Small Animal Imaging System, containing an MT-20 light source (Olympus Biosystems, Planegg, Germany) and DP70 CCD camera (Olympus) were used for imaging GFP-labeled *S. typhimurium* A1-R and orthotopic tumors in live mice [38].

### Detection of GFP-labeled *S. typhimurium* A1-R bacteria in tumors and organs

Tissues from subcutaneous tumors and normal organs (blood, spleen and liver) were removed at termination from the nude mice with subcutaneous tumors. *S. typhimurium* A1-R was extracted from the tumors and organs and cultured in LB agar for 24 hours, and imaged with the Olympus OV100.

### Statistical analysis

PASW Statistics 18.0 (SPSS, Inc) was used for all statistical analyses. The Student's *t*-test was used to compare continuous variables between two groups. Analysis of variance models were used to compare multiple groups. A *p* value of ≤0.05 was considered statistically significant for all comparisons.
